# Sex moderates the association between age and myelin water fraction in the cingulum and fornix among older adults without dementia

**DOI:** 10.3389/fnagi.2023.1267061

**Published:** 2023-12-15

**Authors:** Einat K. Brenner, Katherine J. Bangen, Alexandra L. Clark, Lisa Delano-Wood, Nicole D. Evangelista, Lauren Edwards, Scott F. Sorg, Amy J. Jak, Mark W. Bondi, Sean C. L. Deoni, Melissa Lamar

**Affiliations:** ^1^Department of Psychiatry, University of California, San Diego, La Jolla, CA, United States; ^2^VA San Diego Healthcare System, San Diego, CA, United States; ^3^Department of Psychology, The University of Texas at Austin, Austin, TX, United States; ^4^Department of Clinical and Health Psychology, Center for Cognitive Aging and Memory, College of Public Health and Health Professions, McKnight Brain Institute, University of Florida, Gainesville, FL, United States; ^5^Joint Doctoral Program in Clinical Psychology, San Diego State University/University of California San Diego, San Diego, CA, United States; ^6^Home Base, A Red Sox Foundation and Massachusetts General Hospital Program, Boston, MA, United States; ^7^Bill and Melinda Gates Foundation, Seattle, WA, United States; ^8^Rush Alzheimer’s Disease Center, Rush University Medical Center, Chicago, IL, United States; ^9^Department of Psychiatry and Behavioral Sciences, Rush University Medical Center, Chicago, IL, United States

**Keywords:** myelin, sex, age, myelin water fraction (MWF), fornix (brain), cingulum, memory, neuroimaging

## Abstract

**Background:**

Decreasing white matter integrity in limbic pathways including the fornix and cingulum have been reported in Alzheimer’s disease (AD), although underlying mechanisms and potential sex differences remain understudied. We therefore sought to explore sex as a moderator of the effect of age on myelin water fraction (MWF), a measure of myelin content, in older adults without dementia (*N* = 52).

**Methods:**

Participants underwent neuropsychological evaluation and 3 T MRI at two research sites. Multicomponent driven equilibrium single pulse observation of T1 and T2 (mcDESPOT) quantified MWF in 3 *a priori* regions including the fornix, hippocampal cingulum (CgH), and cingulate cingulum (CgC). The California Verbal Learning Test-Second Edition assessed learning and delayed recall. Multiple linear regressions assessed for (1) interactions between age and sex on regional MWF and (2) associations of regional MWF and memory.

**Results:**

(1) There was a significant age by sex interaction on MWF of the fornix (*p* = 0.002) and CgC (*p* = 0.005), but not the CgH (*p* = 0.192); as age increased, MWF decreased in women but not men. (2) Fornix MWF was associated with both learning and recall (*p*s < 0.01), but MWF of the two cingulum regions were not (*p* > 0.05). Results were unchanged when adjusting for hippocampal volume.

**Conclusion:**

The current work adds to the literature by illuminating sex differences in age-related myelin decline using a measure sensitive to myelin and may help facilitate detection of AD risk for women.

## Introduction

The investigation of white matter demyelination and degeneration in the brain has helped further elucidate the pathophysiological mechanism of both normal aging and neurodegenerative diseases such as Alzheimer’s disease (AD). Previously, many neuroimaging studies of AD have focused on gray matter atrophy thought to be reflective of neurodegeneration more broadly, including beta amyloid (Aβ) and tau tangle accumulation. However, alterations in white matter network topology have been observed in preclinical AD (Fischer et al., 2015), and age-related differences in white matter microstructure that predate gray matter atrophy have been demonstrated using multiple MRI methods (Arshad et al., 2016; Bangen et al., 2021). Also, cognitive decline in normal aging and AD has been associated with changes in several widely distributed cerebral white matter tracts (Delano-Wood et al., 2012; Amlien and Fjell, 2014; Bangen et al., 2021).

Microstructural changes of limbic system regions such as the fornix and cingulum, which have been associated with memory function (Delano-Wood et al., 2012; Van Der Holst et al., 2013; Bangen et al., 2021) remain less understood. Compared to healthy controls, patients with AD show both macrostructural and microstructural white matter alterations across several regions, including the thalamus, thalamic radiation, cingulum, splenium of corpus callosum, and the fornix (Serra et al., 2010). Lower fornix myelin water fraction (MWF)—a measure of myelin content—has been associated with worse memory performance, even after adjusting for hippocampal volume (Metzler-Baddeley et al., 2019; Bangen et al., 2021), and age has been shown to be associated with white matter decline in the fornix, but not the cingulum in healthy older adults (Stadlbauer et al., 2008). A different group observed these same patterns in the fornix and in the inferior, but not superior cingulate bundle (Sullivan et al., 2010). Furthermore, damage to fornix white matter glia contributes to hippocampal gray matter damage in age-dependent limbic decline (Metzler-Baddeley et al., 2019).

Most studies have used diffusion tensor imaging (DTI) to examine microstructural white matter changes. However, this methodology is not specific to myelin and may be associated with a variety of neuropathological metrics such as axonal size, density, and configuration (Beaulieu, 2002). Critically, DTI and myelin water fraction parameters may each offer unique data, and there is therefore increased focus on studying myelin more specifically since its repair and loss has direct implications for the development of white matter abnormalities and associations with Aβ toxicity (Bartzokis, 2011; McAleese et al., 2017). A mutation in oligodendrocytes, which generate myelin, has been associated with white matter damage in AD (Pak et al., 2003), and age-related changes in white matter were also found to be more strongly associated with myelin sheath degeneration than axonal degeneration (Inano et al., 2011). Myelin water fraction has also been shown to be sensitive to age-related changes in white matter, and it increases specificity of measuring myelin content (Faizy et al., 2020).

Although women are at higher risk of developing AD, findings from studies investigating the effect of sex on white matter integrity have been mixed. Some researchers have found minimal or no sex effects in the relationship between age and white matter metrics (Salat et al., 2009; Inano et al., 2011; Faizy et al., 2018). Others have observed differences in the corpus callosum and left-hemisphere regions, where men show greater myelin content than women (Westerhausen, 2003; Liu et al., 2010; Canales-Rodríguez et al., 2021). Sullivan and colleagues noted that men and women showed similar age-related increases in fornix fiber diffusivity, but men showed a significant decline in corpus callosum genu fractional anisotropy compared to women (Sullivan et al., 2010). Most of these studies included young and middle-aged adults. Further research is needed to understand the effect sex may play in the relationship between age and myelin content, particularly in older adults and those at risk for AD.

Decreasing white matter integrity in limbic pathways including the fornix and cingulum have been reported in AD, although underlying mechanisms and potential sex differences remain understudied. Given this, we examined sex as a moderator of the effect of age on MWF, a measure of myelin content, in older adults.

## Materials and methods

### Participants

Fifty-two older adults without dementia were recruited from ongoing aging studies at the University of California, San Diego (UCSD) and University of Illinois Chicago (UIC). Participants were excluded if they had a history of dementia, clinical stroke, neurologic disease (e.g., Parkinson’s disease, multiple sclerosis), head injury with residual cognitive sequelae, or major psychiatric disorder.

Participants underwent clinical interview, brachial artery blood pressure measurement, neuropsychological assessment, and MR exams. Participants at UIC also underwent fasting blood draws. Arterial stiffening was indexed by pulse pressure, which was quantified as systolic minus diastolic pressure. Participants were classified as having diabetes based on self-report, hemoglobin A1c values ≥6.5%, and/or use of an anti-diabetic medication. Self-reported medical history was used to obtain current cigarette smoking, history of cardiovascular disease, history of atrial fibrillation, and current antihypertensive medication use in order to calculate Framingham Stroke Risk Profile (FSRP) score, an index of stroke risk. The updated FSRP provides sex-corrected scores based on age, systolic blood pressure, diabetes, cigarette smoking, cardiovascular disease, atrial fibrillation, and antihypertensive medication use (Dufouil et al., 2017). The study protocol was approved by Institutional Review Board at each institution, and all participants provided informed consent.

### Neuropsychological scores

Episodic memory was assessed by the California Verbal Learning Test – Second Edition (CVLT-II) (Delis et al., 2000). Two metrics were assessed: (1) learning – total learning trials 1–5 and (2) delayed recall – long delay free recall. Standardized scores were adjusted for age and sex (Delis et al., 2001).

### MR image acquisition and analysis

MRI data were acquired on one of two GE 3 T scanners (one at UCSD, the other at UIC). Harmonized MRI acquisition across both scanner sites and analysis methods for this study have been previously described (Bangen et al., 2021). T1-weighted high-resolution anatomical scans were collected at UCSD using a Fast Spoiled Gradient Recall acquisition (172 1 mm × 0.977 mm × 0.977 mm contiguous sagittal slices, field of view [FOV] = 25 cm, repetition time [TR] = 8 ms, echo time [TE] = 3.1 ms, flip angle = 12, inversion time [T1] = 600 ms, 256 × 192 matrix, Bandwidth = 31.25 kHz, frequency direction = S-I, NEX = 1). T1-weighted high-resolution anatomical scans were collected at UIC using Brain Volume (BRAVO) imaging sequence (120 interleaved axial slices, FOV = 22 mm^2^; TR/TE = 1,200 ms/5.3 ms). The T1-weighted scans collected at UCSD had dimensions of 1.0 mm x 0.977 mm x 0.977 mm. The T1-weighted scans collected at UIC had dimensions of 1.5 mm x 0.43 mm x 0.43 mm. T1-weighted images at both sites were processed with FreeSurfer 6.0 (Dale et al., 1999; Fischl et al., 2002). Volumetric data, including hippocampal volume, was derived using FreeSurfer 6.0 and visually inspected. Normalized hippocampal volume was calculated by dividing by total intracranial volume.

**Figure 1:**
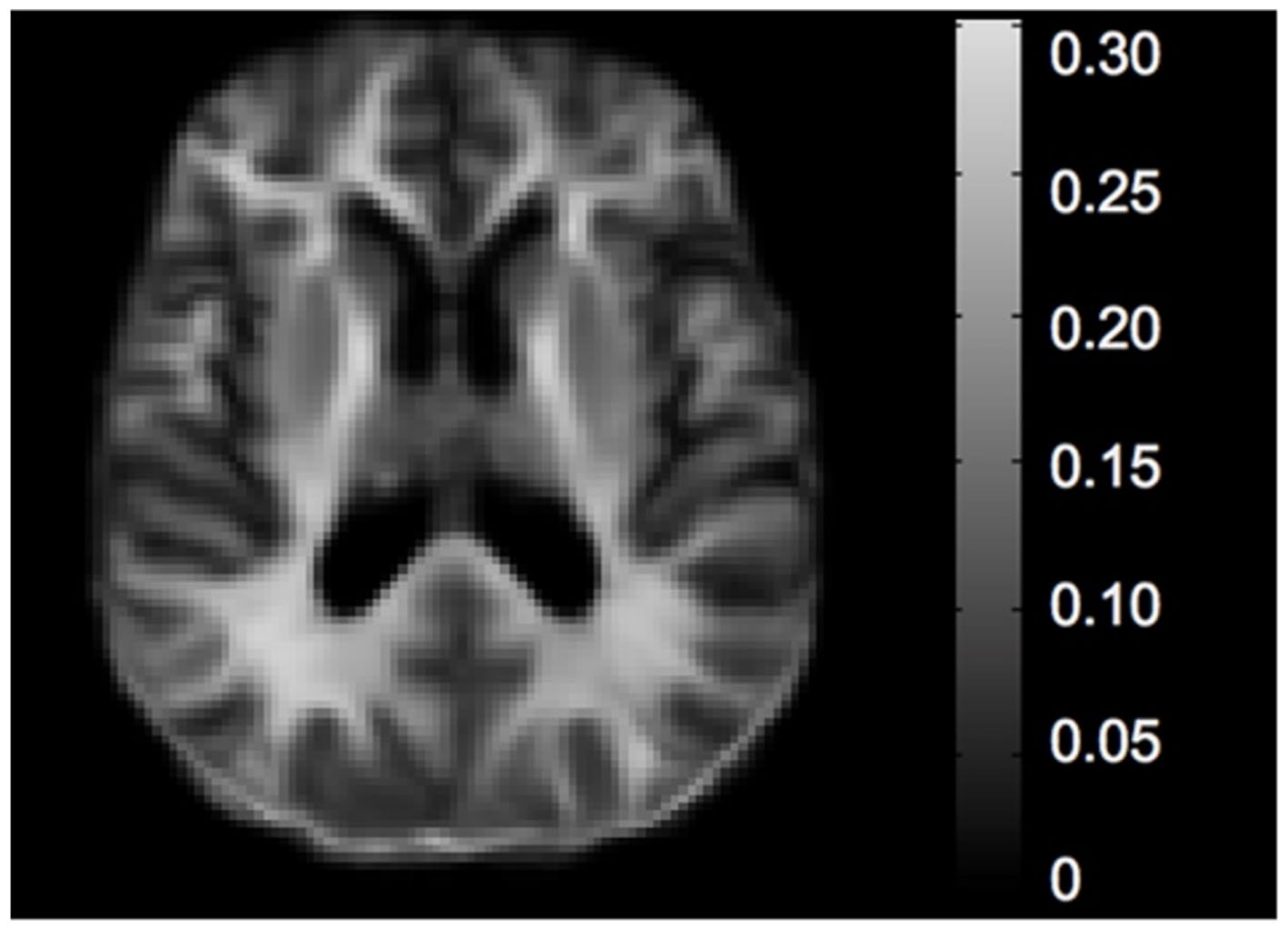
Sample MWF map.

For the mcDESPOT sequence, a series of spoiled gradient recalled echo were acquired (SPGR; TR = 5.3 ms, TE = Min Full, flip angle = 18, FOV =24.0) and T2/T1-weighted balanced steady-state free precession (SSFP) data over a range of flip angles (Deoni, 2011). To correct for B1 inhomogeneities, we collected an inversion-recovery prepared SPGR (IR-SPGR) scan (TR = 5.3 ms, TE = Min Full, flip angle = 60, field of view = 24.0) and SSFP phase 0 (TE = Min Full, flip angle = 60, FOV = 24.0) with two phase-cycling patterns to correct for main magnetic field (B0) off-resonance effects. Following acquisition, the SPGR and SSFP images comprising each participant’s dataset were linearly coregistered to account for subtle intrasession head movement (Jenkinson et al., 2002). We obtained MWF maps (Figure 1) by fitting SPGR and bSSFP data to a three-pool model that included two exchanging water pools (myelin water and water both inside and outside the axon) as well as a third non-exchanging free water pool (Deoni et al., 2012). MWF map voxel dimensions were approximately 1.7 mm^3^ isotropic.

Post-processing of mcDESPOT data was completed using the Oxford Centre for Functional Magnetic Resonance Imaging of the Brain (FMRIB) Software Library (FSL) (Smith et al., 2004). T1-weighted images were downsampled to the 2mm^3^ resolution of the MNI152 template. Brain Extraction Tool (BET) was used to remove non-brain voxels from images. We then used FMRIB’s Linear Image Registration Tool (FLIRT) (Jenkinson et al., 2002) and FMRIB’s Nonlinear Image Registration Tool (FNIRT) to linearly and then non-linearly register T1-weighted images and mcDESPOT myelin volume fraction images to the MNI152 T1 2mm resolution brain template. FSL’s Automated Segmentation Tool (FAST) was applied to segment T1-weighted images into white matter, gray matter, and cerebrospinal fluid components. The resulting FNIRT transforms were then applied to the myelin volume fraction masks. We multiplied segmented white matter masks by ROI masks to ensure inclusion of exclusively white matter voxels prior to extracting regional MWF values. The ROIs applied to the mcDESPOT data were selected using the ICBM-DTI-81 stereotaxic white matter parcellation map and included the fornix, cingulate cingulum (CgC), and hippocampal cingulum (CgH). Visual inspection of each participant’s T1-weighted image, MWF map, and the MNI152 template showed good alignment.

### Statistical analyses

Prior to analyses, data were examined for violations of assumptions of the statistical procedures employed including posterior predictive check, linearity, homogeneity of variance, influential observations, collinearity, and normality of residuals. There were no outliers greater or less than three standard deviations away from the mean in any of the independent and dependent variables.

First, we used linear regression models to examine two-way interactions between age and sex on MWF of 3 *a priori* regions (fornix, CgC, and CgH) as the dependent variable (Table 1). Each region was examined in a separate model. These models included covariates of site and pulse pressure, main effects of age and sex, and the two-way interaction of age and sex. To interpret significant interactions, we then stratified by sex and ran linear regression models examining the associations between age and regional MWF (adjusting for site and pulse pressure) separately for women and men.

**Table 1:** Regression models examining interactive effects of age and sex on regional myelin water fraction.

	Fornix					Cingulate cingulum	Hippocampal cingulum
	Estimate	*β*	S.E.	*t*	*p*	Estimate	*β*	S.E.	*t*	*p*	Estimate	*β*	S.E.	*t*	*p*
Age	−4.57E-04	−0.21	3.07E-04	−1.49	0.143	−0.001	−0.24	0.001	−1.84	0.072	−0.001	−0.18	0.0004	−1.25	0.218
Sex	−3.91E-03	−0.15	3.59E-03	−1.09	0.282	−0.01	−0.29	0.01	−2.33	**0.024**	−0.009	−0.23	0.005	−1.65	0.105
Site	−5.66E-03	−0.20	4.02E-03	−1.41	0.17	−0.01	−0.26	0.01	−2.06	**0.045**	−0.001	−0.03	0.006	−0.22	0.829
Pulse pressure	1.93E-06	0.002	1.27E-04	0.015	0.988	−0.0003	−0.20	0.0002	−1.63	0.111	−0.0001	−0.11	0.0001	−0.78	0.439
Age x sex	−1.74E-03	−4.93	5.83E-04	−2.99	**0.005**	−0.003	−4.80	0.001	−3.32	**0.002**	−0.001	−2.38	0.001	−1.33	0.192

This table shows the results of regression models examining two-way interactions between age and sex on the 3 a priori regions, examined in separate models. These models adjusted for site and pulse pressure. Bold values are statistically significant, *p* < 0.05.

Second, we examined associations between MWF in the *a priori* regions of interest and each memory measure (i.e., learning and recall) as dependent variables in separate models adjusting for collection site, pulse pressure, age, and sex. We first ran the models across the entire sample, and then these models were stratified by sex for further interpretation.

For all models, we ran secondary analyses additionally adjusting for normalized hippocampal volume. We also ran all primary analyses not adjusting for pulse pressure, and the results remained the same. The only result that changed was that, additionally adjusting for normalized hippocampal volume, there was a significant association between age and MWF on fornix in men. All analyses were conducted using R Statistical Software (R Core Team, 2023) with significance set at *p* < 0.05.

## Results

### Participant characteristics

Characteristics of each group can be viewed in Table 1. The men and women did not differ by average age, education, collection site, or race, *p* > 0.05. There were also no significant differences in sex distribution, age, fornix MWF, or CgH MWF between the collection sites, *p* > 0.05. *T*-tests showed a significant difference between CgC MWF between the collection sites (UIC M = 0.11, UCSD M = 0.09, *p* = 0.027).

### Two-way interactions between age and sex on myelin water fraction

**Figure 2:**
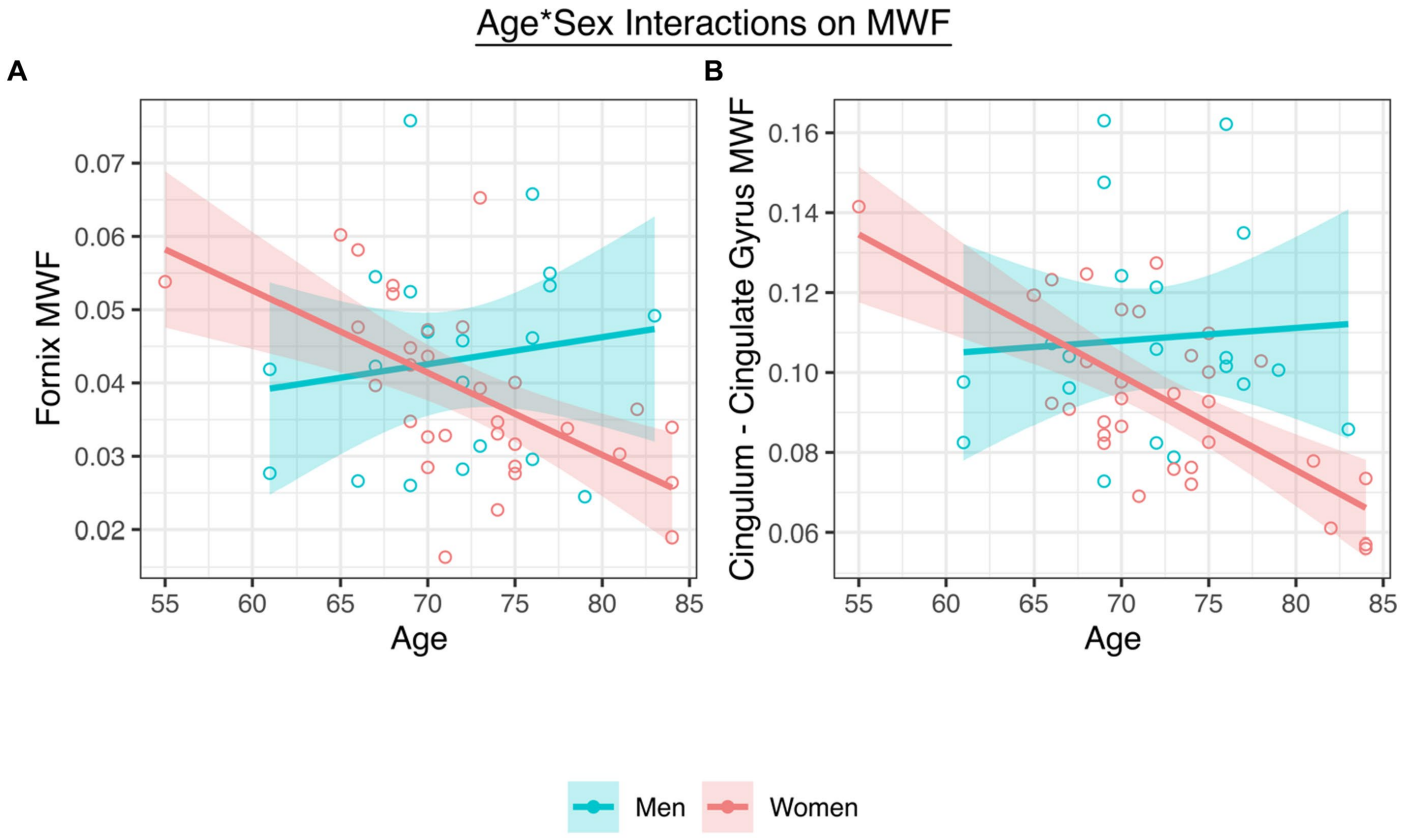
**(A)** A significant interaction between age and sex on fornix MWF. **(B)** A significant interaction between age and sex on CgC MWF.

Adjusting for collection site and pulse pressure as well as the main effects of age and sex, there was a significant interaction between age and sex on fornix MWF (*t* = −2.99, *p* = 0.005, *β* = −4.93; Figure 2A; Table 2) as well as on CgC MWF (*t* = −3.32, *p* = 0.002, *β* = −4.80; Figure 2B). The interaction between age and sex on CgH MWF was not significant (*t* = −1.33, *p* = 0.192, *β* = 0.08).

**Table 2:** Participant characteristics.

	Men (*N* = 20)	Women (*N* = 32)	Between-group differences
Demographics
Age	71.6 ± 5.73	72.4 ± 6.24	*t* = −0.48, *p* = 0.64, *d* = 0.13
Education	17.40 ± 2.19	16.56 ± 2.03	*t* = 1.38, *p* = 0.18, *d* = 0.40
Collection Site	35% UIC; 65% UCSD	22% UIC; 78% UCSD	*X*^2^ = 0.51, *p* = 0.47, *V* = 0.03
Race	80% White, 15% Black, 5% Asian	81% White, 13% Black, 6% Asian	*X*^2^ = 0.09, *p* = 0.96, *V* < 0.00
Vascular Health
Pulse Pressure	56.73 ± 11.05	54.11 ± 15.77	*t* = 0.70, *p* = 0.49, *d* = 0.19
Diabetes	15% with diabetes, 85% without diabetes	13% with diabetes, 87% without diabetes	*X*^2^ = 0.07, *p* = 0.80, *V* = 0.04
Current Smoking	15% smokers, 85% nonsmokers	3% smoker, 97% nonsmokers	*X*^2^ = 2.44, *p* = 0.12, *V* = 0.22
FSRP	7.56 ± 3.34	8.21 ± 6.26	*t* = −0.49, *p* = 0.63, *d* = 0.12
Memory
List Learning	57.85 ± 12.78	58.81 ± 13.13	*t* = −0.26, *p* = 0.80, *d* = 0.07
Delayed Recall	0.32 ± 1.41	0.39 ± 1.03	*t* = −0.19, *p* = 0.85, *d* = 0.06

“Age” and “Education” are presented in years. “Pulse pressure” was quantified as systolic minus diastolic blood pressure and is presented in units of millimeters of mercury (mmHg). Two-sample t-tests were used for evaluating differences in continuous variables between men and women. Chi-square tests were used to evaluate differences in categorical variables between men and women. “V” indicates Cramer’s V. “d” indicates Cohen’s d. “UIC” indicates University of Illinois Chicago, and “UCSD” indicates University of California, San Diego. “FSRP” indicates Framingham Stroke Risk Profile 10-year stroke probability as a percent. California Verbal Learning Test – Second Edition (CVLT-II) List Learning standardized scores are T-scores, and CVLT-II Delayed Recall standardized scores are z-scores.

To interpret the significant interactions, analyses stratified by sex revealed that women (fornix *t* = −3.40, *p* = 0.002, *β* = −0.51; CgC t = −4.71, *p* < 0.001, *β* = −0.59), but not men (fornix *t* = 0.94, *p* = 0.423, *β* = 0.21; CgC t = 1.47, *p* = 0.160, *β* = 0.37), showed significant age-associated declines in MWF.

In a secondary analysis, all results were unchanged after additionally adjusting for normalized hippocampal volume. That is, there was a significant interaction between age and sex on fornix MWF (*t* = −3.12, *p* = 0.003, *β* = 0.43) as well as on CgC MWF (*t* = −3.25, *p* = 0.002, *β* = 0.34). The interaction between age and sex on CgH MWF remained non-significant (*t* = −1.19, *p* = 0.239, *β* = 0.14). In interpreting the significant interactions through regression models stratified by sex, the relationships between age and MWF remained significant in women (fornix *t* = −2.73, *p* = 0.011, *β* = −0.40; CgC t = −4.13, *p* < 0.001, *β* = −0.55) but not men (fornix *t* = 1.96, *p* = 0.069, *β* = 0.40; CgC t = 2.01, *p* = 0.063, *β* = 0.49).

### Associations between regional MWF and memory

Across all participants, adjusting for age, sex, pulse pressure, and collection site, fornix MWF was associated with measures of learning (*t* = 3.41, *p* = 0.001, *β* = 0.47) and recall (*t* = 2.88, *p* = 0.006, *β* = 0.43). Stratifying by sex, associations between fornix MWF and learning were significant in both men (*t* = 2.51, *p* = 0.024, *β* = 0.60) and women (*t* = 2.78, *p* = 0.010, *β* = 0.60), and the association between fornix MWF and recall was significant in women (*t* = 2.93, *p* = 0.007, *β* = 0.64), but not men (*t* = 1.80, *p* = 0.093, *β* = 0.51). MWF of the two cingulum regions were not associated with either learning or recall (*p*’s > 0.05).

In a secondary analysis additionally adjusting for normalized hippocampal volume, significant associations remained between MWF and both learning (*t* = 2.30, *p* = 0.027, *β* = 0.36) and recall (*t* = 2.06, *p* = 0.046, *β* = 0.36). Stratifying by sex, the associations between fornix MWF and both learning and recall remained significant in women (learning *t* = 2.30, *p* = 0.031, *β* = 0.55; recall *t* = 2.71, *p* = 0.012, *β* = 0.66), but not in men (learning *t* = 0.94, *p* = 0.364, *β* = 0.28; recall *t* = 0.50, *p* = 0.624, *β* = 0.18).

## Discussion

Our results demonstrate that sex moderates the associations between age and MWF in both the fornix and the CgC, but not the CgH. Specifically, the significant interactions showed that for women, but not men, as age increased, MWF decreased. We also examined associations between MWF in each of the three regions and both learning and delayed recall. Results showed that fornix MWF was associated with learning regardless of sex and was associated with delayed recall only in women. MWF of the two cingulum regions were not associated with either cognitive measure regardless of sex.

Our findings suggest that, in older adults, the association between age and MWF of both the fornix and CgC is dependent on sex, even after adjusting for several relevant risk factors. Notably, these interactions showed that for women, as age increased, fornix and CgC MWF decreased, but for men, the relationship between age and MWF was not significant. Age-related white matter decline in the fornix and CgC have been observed in previous studies (Stadlbauer et al., 2008; Bastin et al., 2010; Fletcher et al., 2013), but few have stratified by sex and even fewer have investigated MWF using multicomponent relaxometry techniques. Thus, while some (Bastin et al., 2010) but not all (Kodiweera et al., 2016) studies have reported sex differences in DTI-derived white matter alterations, ours is the first to examine this relationship as it relates to myelin integrity. Furthermore, although previous work has shown age-related alterations in the fornix (Bastin et al., 2010), ours is the first to carefully consider sex in tandem with age. Interactions between age and sex were not significant when examining the CgH suggesting that the fornix and CgC may be a more sensitive marker of age-related demyelination and other neurodegenerative changes in the brain (Gazes et al., 2019; Metzler-Baddeley et al., 2019). Taken together, our results suggest that women exhibit more age-related demyelination in brain regions critical for learning and memory than their male counterparts.

Results also showed significant associations between learning and fornix MWF for women and between recall and fornix MWF across both groups, and these relationships remained significant in women, but not men, even after adjusting for hippocampal volume. However, the associations between CgC or CgH MWF and learning/recall were not significant. These results are in line with research showing that fornix, but not hippocampal, integrity is associated with performance on a memory test (Gazes et al., 2019). Furthermore, volume of the fornix has been shown to be a stronger predictor of cognitive decline among the cognitively normal than hippocampal volume (Fletcher et al., 2013). Also, although decreased cingulum white matter integrity has been observed in individuals with amnestic mild cognitive impairment or AD (Gozdas et al., 2020; Luo et al., 2020; Wong et al., 2020), our sample of older adults without dementia may be too early in the aging or disease process to detect these changes. Future work using MWF should examine these associations in older adults with AD, as well as middle-aged adults, to further elucidate the timeline of its effect on cognition across the AD spectrum.

There are several limitations to our study worth noting. First, our study is preliminary given its small sample size and cross-sectional design. Longitudinal studies with larger samples are needed to further clarify the role that sex plays in limbic system myelin integrity across the aging spectrum. Our sample was also mostly comprised of White and relatively highly educated individuals. The relationships between age, sex, and myelin content may be different in a more diverse sample or one with greater variability in education or socioeconomic status. Additionally, although defined as such in this study, sex is not necessarily a binary construct, and we did not evaluate intersex. We also did not have body mass index or cholesterol data for all participants, and these are factors that can impact white matter integrity. Our analyses were also limited to one shared memory measure between the two collection sites. Finally, the myelin-related signal can be influenced by physiological factors such as white matter injury and inflammation as well as data acquisition factors such as flip angle errors, which may reduce the precision of individual MWF estimates. In the present study, we visually inspected all data to ensure adequate quality data. Future large-scale studies should employ multiple measures of learning and memory in order to more comprehensively evaluate both verbal and visual memory measures as they relate to myelin metrics in at-risk older adults.

## Conclusion

Results of our study suggest that the relationship between age and myelin content of limbic fiber pathways depends on sex, with women showing age-related decreases that are associated with poorer memory performance. We also found significant associations between learning/recall and fornix MWF in women. The current work adds to the literature by illuminating the role that sex plays in age-related myelin decline using a more sensitive measure of myelin content. Understanding sex differences in MWF may also facilitate earlier detection of AD risk for women. Future work will expand on these findings in a larger, longitudinal sample, such as whether baseline fornix MWF is differentially associated with memory decline in men versus women. Future analyses may also examine whether age interacts with sex to affect change in myelin content of the limbic fiber pathways.

## Data availability statement

The raw data supporting the conclusions of this article will be made available by the authors, without undue reservation.

## Ethics statement

The studies involving humans were approved by University of California San Diego IRB and the University of Illinois Chicago IRB. The studies were conducted in accordance with the local legislation and institutional requirements. The participants provided their written informed consent to participate in this study.

## Author contributions

EB: Formal analysis, Methodology, Visualization, Writing – original draft, Writing – review & editing. KB: Conceptualization, Data curation, Formal analysis, Funding acquisition, Investigation, Methodology, Project administration, Resources, Supervision, Writing – original draft, Writing – review & editing. AC: Writing – review & editing. LD-W: Writing – review & editing. NE: Writing – review & editing, Data curation. LE: Writing – review & editing. SS: Writing – review & editing, Data curation, Methodology. AJ: Writing – review & editing, Conceptualization. MB: Writing – review & editing. SD: Methodology, Writing – review & editing. ML: Conceptualization, Data curation, Funding acquisition, Investigation, Project administration, Resources, Supervision, Writing – original draft, Writing – review & editing, Formal analysis, Methodology.
